# Distal weight bearing in transtibial prosthesis users wearing pin suspension

**DOI:** 10.3389/fresc.2023.1322202

**Published:** 2023-12-21

**Authors:** Adam J. Krout, Mathew J. Weissinger, Joseph C. Mertens, Katheryn J. Allyn, Brian G. Larsen, Nicholas K. McCarthy, Joseph L. Garbini, Joan E. Sanders

**Affiliations:** ^1^ Sanders Prosthetic and Orthotic Science & Technology Laboratory, Bioengineering Department, University of Washington, Seattle, WA, United States; ^2^ Mechanical Engineering Department, University of Washington, Seattle, WA, United States

**Keywords:** prosthetics, residual limb, distal weight bearing, transtibial amputee, socket fit, pin suspension, limb-socket interface, end bearing

## Abstract

**Introduction:**

Low-level distal weight bearing in transtibial prosthesis users may help maintain perfusion and improve both proprioception and residual limb tissue health.

**Methods:**

The primary objectives of this research were to develop a sensor to continuously measure distal weight bearing, evaluate how prosthesis design variables affected weight bearing levels, and assess fluctuations in distal weight bearing during at-home and community use.

**Results:**

In-lab testing on a small group of participants wearing adjustable sockets demonstrated that if distal contact was present, when socket size was increased distal weight bearing increased and when socket size was reduced distal weight bearing decreased. During take-home use, participants accepted the distal weight bearing level set by the research team. It ranged between 1.1% and 6.4% BW for all days tested. The coefficient of variation (standard deviation/mean) ranged from 25% to 43% and was expected due in part to differences in walking style, speed, terrain, direction of ambulation, and bout duration. Two participants commented that they preferred presence of distal weight bearing to non-presence.

**Discussion:**

Next steps in this research are to develop clinical practices to determine target distal weight bearing levels and ranges, and to simplify the design of the sensor and weight bearing adjustment mechanism for clinical use.

## 1. Introduction

Distal weight bearing, force transferred from the bottom of the residual limb to the socket, is relevant to the well-being of a person with transtibial amputation. Some distal weight bearing may generate a pumping effect on the residual limb during walking that helps maintain perfusion and reduce edema (1). Research from the zoology and evolutionary biology literature suggest that soft tissue will adapt its collagen architecture to better tolerate mechanical stress if subject to low to moderate cyclic loads (2). Excessive distal weight bearing may put residual limb tissues at risk of injury. Prosthetists use primarily two techniques during office visits to assess distal weight bearing: (i) A putty ball is placed in the bottom of the socket and its thickness before and after weight bearing is compared. (ii) The color of distal soft tissue after walking is assessed—a bright red color may indicate excessive distal pressure and the need for an adjustment.

In previous studies, researchers measured distal weight bearing in participants with transtibial amputation (3–5). Katz et al. (3) assessed distal weight bearing by isolating the distal section of the socket and instrumenting it with a load cell. Different thickness spacers were placed underneath the distal section to shorten the socket and increase the force on the distal residual limb. Testing was conducted on a group of six participants 27–67 years old who regularly used a patellar tendon bearing socket with strap suspension and a Pelite™ liner. Participants' applied force to the distal end of their socket while weight bearing just under their pain threshold was a median of 30% (range 13%–48%) of their maximum vertical ground reaction force when they were walking and a median of 30% (range of 11%–55%) of their maximum vertical ground reaction force when they were standing. Persson and Liedberg (4) conducted testing on 85 participants with transtibial amputation (mean age 67 years). Participants' residual limb maximum weight bearing during standing was measured using a fitting stool equipped with a curved top surface to support the residual limb. No socket was worn. Participants' residual limb maximum weight bearing without pain during standing was a mean of 17.2% (SD 13.1) of their body weight (BW). Participants with diabetes tolerated a greater force (mean 21.5% BW) than participants without diabetes (mean 14.3% BW) to a significance level of 0.05. Participants with a residual limb that was rounded at the distal end demonstrated a lower residual limb weight bearing force (mean 16.2% BW) than participants with a pointed distal end (mean 18.5% BW) but the difference was not statistically significant. Rich et al. (5) inserted a pneumatic pressure sensor into the distal end of the socket in six participants with transtibial amputation using sleeve suspension. The sensor data were not quantified into units of force though a relative change in the data within an in-lab test session for different sock thicknesses was demonstrated.

This research is directed towards a novel instrument to clinically evaluate if low-level distal end bearing is beneficial to participants with transtibial amputation using sockets with locking pin suspension. The instrument sensed pin vertical position and residual limb distal weight bearing. We manually adjusted the system to achieve different weight bearing levels. A small group of participants with transtibial amputation wore the device during a structured in-lab protocol to determine if distal weight bearing increased when socket size was increased and decreased when socket size was decreased. Then the instrumentation was improved to simultaneously collect liner-to-socket distance data from sensors embedded in the socket so that relationships between limb-socket position and distal limb motion could be explored. Participants wore the test prosthesis in their free-living environment for approximately one week. The daily standard deviation in the data was calculated, and plots of sensed distance against pin height were generated. The instrument may be a clinically useful platform for measuring how prosthesis design variables and participant characteristics affect limb motion and distal weight bearing, and potentially allow for an automatically adjusting socket that controls distal weight bearing to clinically beneficial levels. The technology is intended to support patient education, clinical diagnostics, and treatment design to optimize prosthetic fit for individual patient care.

## 2. Materials and methods

### 2.1. Participant inclusion and exclusion criteria

Participants were included in this study if they were at least 18 years of age, had a transtibial amputation at least 18 months prior, were using a definitive prosthesis, were at a MFCL level (6) of K2 (had the ability or potential for ambulation with the ability to traverse low-level environmental barriers such as curbs, stairs, or uneven surfaces) or higher, and had participated in a study wearing a three-panel motor-driven adjustable socket (7). The socket from that study needed to be of acceptable fit as deemed by the research prosthetist. This last criterion was necessary to ensure that a properly fitting adjustable socket was available for use in the present study. Additional inclusion criteria were that participants self-reported walking at least 7 h/wk and were capable of continuous walking on a treadmill for at least 8 min. Participants were not included if they used a walking aide (e.g., cane, walker) or were currently experiencing residual limb skin injury.

### 2.2. Sensors and sockets

Locking pin suspension is a means for maintaining proper position of the residual limb in the socket during the swing phase of gait. A rastered pin with about 7 notches that fastens to the bottom of the liner is inserted into a ratchet in the bottom of the socket. The ratchet holds the pin at the deepest pin notch reached during ambulation. The participant may release the ratchet via a simple mechanism accessible on the side of the socket.

A sensor that measured the depth of the locking pin within the shuttle lock, developed in our prior work (8), was re-designed to incorporate springs into a platform that supported the locking pin. This addition allowed the sensor's measurement of pin position to be converted to measurement of applied force by multiplying by the stiffness of the springs. The force applied through the locking pin was measured continuously during prosthesis use.

**Figure 1:**
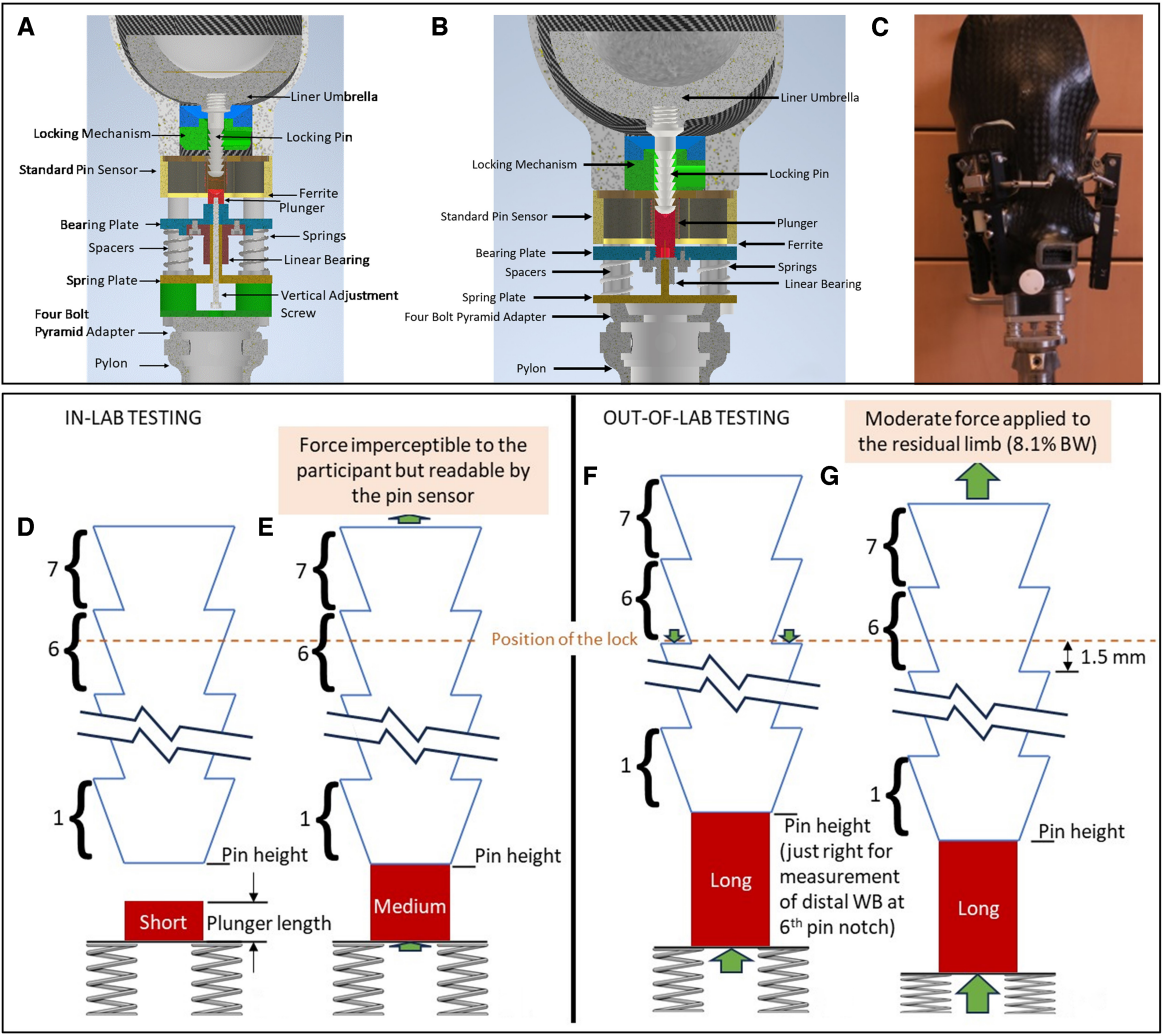
(**A**–**G**) instrumentation. (**A**) Design used for in-lab testing. The resting pin height was adjusted by turning the “Vertical adjustment screw” using an Allen key. The pylon had to be removed to access the Allen key. This version of the sensor weighed 326.4 g. (**B**) Design used for take-home testing. The pin height was adjusted by selecting a plunger length that caused the springs to engage at 0.1 mm before the pin entered the participant's normal pin notch. This version of the sensor weighed 241.0 g. (**C**) Instrumented socket ready for take-home use. An adjustable-size socket with motor-driven panels was used in this study. In **D**–**G**, the locking pin (solid blue line), plunger (red), and springs (gray) are shown. The dashed orange line indicates the position of the pin lock, and the green arrows show force applied to the locking pin and plunger in the four states (**D**–**G**). The *1*, *6*, and *7* indicate the pin notch. Images (**D**) and (**E**) reflect conditions during the in-lab test protocol, and (**F**) and (**G**) reflect conditions during the out-of-lab protocol. (**D**) Using a short plunger, the participant does not displace deep enough into the socket during stance phase to contact the top of the plunger and thus no distal weight bearing force is applied. (**E**) Using a medium plunger, the participant lightly contacts the plunger during stance phase. The resistance from the springs applies a very low distal weight bearing force imperceptible to the participant but readable by the pin sensor. (**F**) The long plunger length pushes up the locking pin such that resistance from the springs applies a force through the pin just as it transitions to the 6th pin notch, this participant's usual pin notch. The force is 0.1 mm × 38.4 N/mm = 3.8 N = 0.5% BW for a 73 kg (160 lb) person. Because the lock holds the pin at this position, no force is applied to the residual limb until weight bearing is ≥3.8 N. (**G**) The participant distal weight bearing during stance phase causes the pin to move down 1.50 mm from (**F**), applying an additional force of 1.50 mm×38.4 N/mm = 57.6 N, a total of 61.4 N (13.8 lb), corresponding to an 8.6% BW force for a 73 kg (160 lb) person.

Four springs were placed on extensions of the four posts that connected the socket to the pyramid adapter of a transtibial prosthesis. The springs provided an elastic element that supported the locking pin via a spring plate and plunger as shown in Figure 1A. A vertical adjustment screw passing through a linear bearing was threaded into the spring plate such that adjustment of the screw extended the plunger, changing the distance between the springs and pin (termed the *plunger length* in Figure 1D), drawing it closer to contact with the bottom of the locking pin. A concept diagram of how adjustment of the plunger length affected the height of the locking pin and the compression of the springs is shown in Figures 1D–G. Plunger lengths ranging from 0.0 to 10.0 mm were tested in this study. The screw for adjustment was accessed through a hole in the bottom of the baseplate while the prosthesis was doffed. The initial design (Figure 1A) was used during an in-lab structured protocol described below.

A more compact design (Figure 1B) that eliminated the vertical adjustment screw and applied force directly to the plunger was used for out-of-lab tests. In this design, different plunger lengths were selected for each user so that the springs started to be compressed just above the transition to the lowest serration in the ratchet, i.e., the participant's deepest pin notch (Figure 1F). Physically, during weight bearing at this plunger length, the plunger contacted the bottom of the pin, the springs further compressed (Figure 1G), and the pin displaced deeper into the pin sensor causing a change in the sensed pin height. Because the vertical adjustment screw was removed for the out-of-lab design, the height of the system was reduced from 77 mm to 42 mm. The out-of-lab pin sensor weighed 78.0 g, and the assembly underneath weighed 163 g, an overall reduction of 85.4 g from the initial design shown in Figure 1A. To calibrate the pin sensor, a bench test jig was used to position the pin at known heights above the sensor (Supplementary Presentation S1).

An in-lab study protocol was designed to collect data on transtibial prosthesis users at different plunger lengths and at different socket sizes. A motor-driven adjustable socket was used to set the socket size (Figure 1C). The test sockets were created for the study participants as part of a prior research investigation (7). The test sockets were equipped with three adjustable panels positioned at anterior medial, anterior lateral, and posterior mid-limb locations. Motors fastened to frames on the outside of the socket drove a screw-driven winch mechanism, moving the panels radially inward and outward. The winch was attached to a rod that passed through the back side of the panel, allowing the panel to rotate about an axis parallel to the socket surface in the transverse plane (Figure 1C). This design avoided panel protrusion into the residual limb at the top or bottom of the panel. No padding was necessary on the insides of the panels nor was a flexible inner socket needed. Panel radial motion was controlled via wireless commands sent from a phone app.

The test sockets were duplicate in shape to participants' regular sockets. To make the test sockets, we measured the shapes of participants' regular sockets using a high-resolution coordinate measurement machine (FARO Arm Platinum, Lake Mary, FL). Each socket was fabricated with six thin inductive sensor antennae placed within the socket wall. Sensors were positioned at anterior proximal, anterior mid-limb, anterior distal, posterior lateral mid-limb, posterior medial mid-limb, and posterior distal locations as shown in Supplementary Presentation S2, using methods detailed in prior work (9). The sensors measured the distance to a trace amount of iron powder placed in the elastomeric liner just under the fabric backing. The liners were purchased from a prosthetics manufacturer contracted to make the liners for research purposes (WillowWood, Mt. Sterling, OH). Participants wore the ferrous liner during both the in-lab and out-of-lab test sessions.

A standard 18.5-mm length locking pin with seven notches was used in all tests since this length ensured that the pin sensor data were within range. Notch 1 engaged the locking mechanism. At the highest pin notch during calibration, typically notch 7, the pin was at the minimum pin height, which was defined as the 0.00 mm pin height.

Bench tests were conducted to evaluate the spring constant of the four-spring support system. Three different sets of four springs were tested. Different spring stiffnesses were expected to be necessary to accommodate different participant weights to keep the pin within a proper displacement range during ambulation and maintain sufficient sensitivity. The pin sensor and four-spring support system was fastened to the base of a unidirectional testing machine (model 5944, Instron, Norwood, MA), and a controlled crosshead displacement was applied. The displacement rate was 3 mm/min, and both loading and unloading tests were conducted. The pin height was plotted against force, and the spring constant was determined using a least-squares fit to the data.

### 2.3. In-lab testing protocol

The purposes of the in-lab study were to evaluate if increasing socket size (using adjustable panels on the socket) introduced a measurable decrease in pin height and to identify the minimum plunger length at which distal weight bearing was introduced. Only pin sensor data and not limb-socket distance sensor data were collected.

**Figure 2:**
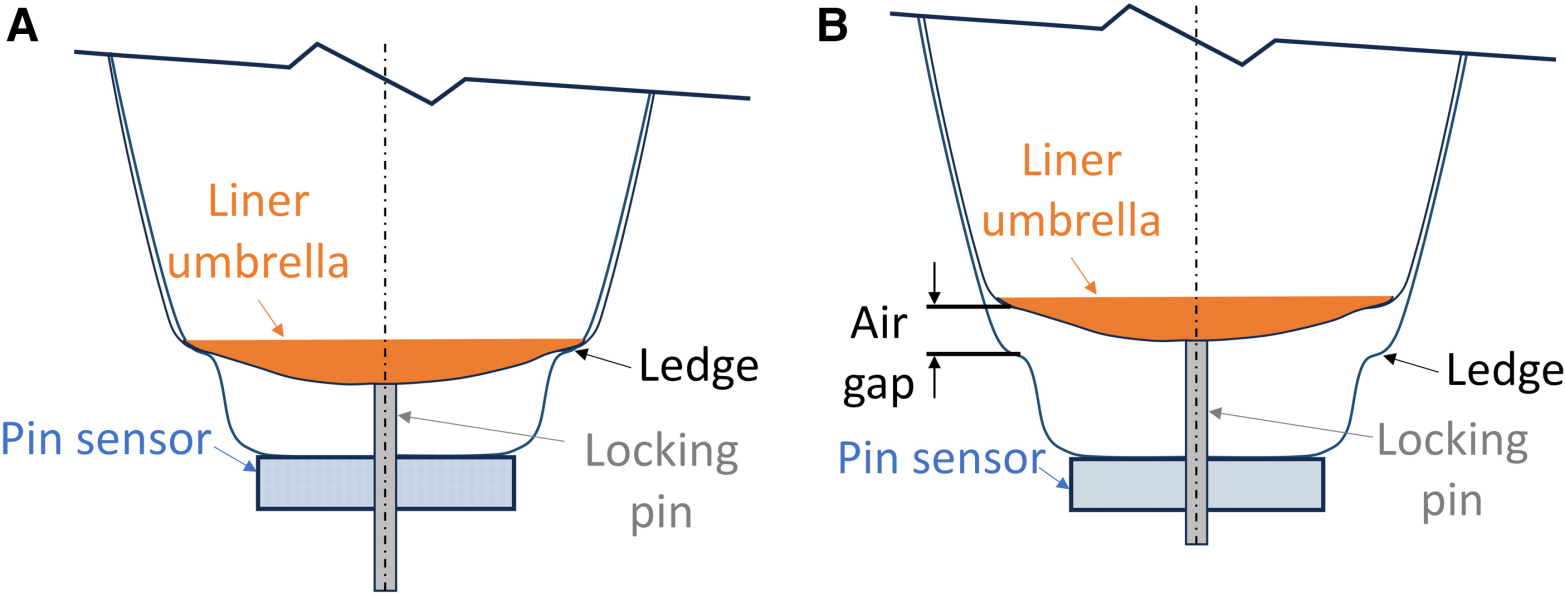
(**A**) Diagram showing the lowest possible pin height. The liner umbrella is at its lowest possible position. There is no air gap between the liner umbrella and the ledge. (**B**) Diagram showing an air gap between the liner umbrella and the ledge. The ledge is formed by the technician during socket fabrication to ensure that the liner does not move down excessively into the socket.

Before the participant arrived, the liner to be worn was inserted and pushed into the bottom of the socket (no residual limb) to record the minimum possible pin height. The minimal possible pin height was at the deepest possible location of the pin at full insertion for the liner-socket combination being used. For all three participant sockets, at the minimum possible pin height the outer edge of the umbrella contacted a ledge on the side of the socket that prevented further downward displacement leaving an air gap (Figure 2A) between the bottom of the umbrella and the ledge made in the socket during fabrication to prevent excessive downward displacement of the liner. The diameter of the relatively stiff part of the umbrella on the bottom of the liner was measured using calipers so that later the distal weight bearing force measurement could be converted to distal tissue pressure. To determine the appropriate range of panel positions to test, each participant performed an initial walk where the panels were incrementally loosened in 0.20-mm radial increments using a phone app that controlled the motors (10, 11). The user's self-reported maximum acceptable socket size was identified. The panels were incrementally tightened, and the user's self-reported minimum acceptable socket size was identified. These two positions were designated as the loosest and tightest socket sizes, respectively. The midpoint between them was termed the midpoint size.

**Figure 3:**
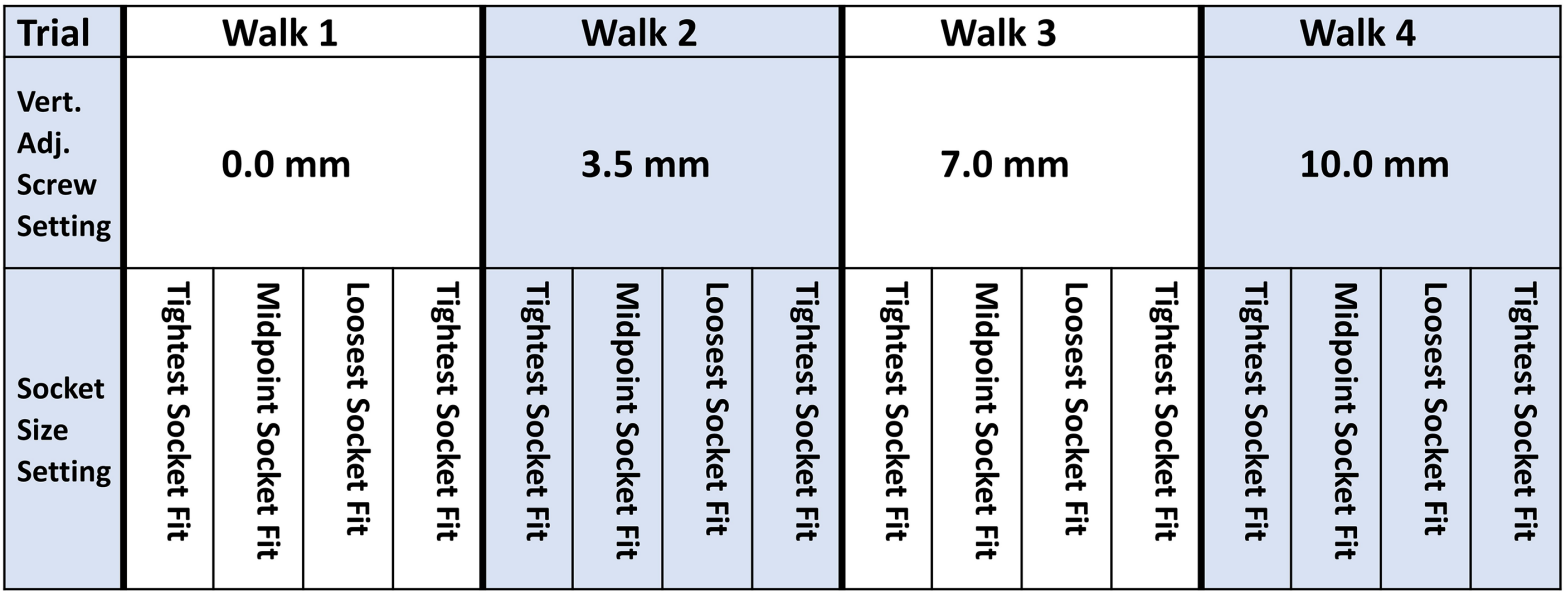
In-Lab testing protocol. Vertical adjustment (Vert. Adj.) screw setting changes were made in between the four 8-min walks while the socket was doffed. Socket size adjustments were made every 2 min while participants walked on the treadmill.

Participants performed four 8-min walks separated by approximately 4-min sits to change the plunger length (Figure 3). During each 8-min walk, the panels were initially positioned at their tightest setting. The panel position was changed at 2-min intervals to midpoint, loosest, and back to tightest. During each walk, participants were queried if the setting caused discomfort. This 8-min walk cycle was repeated four separate times with the plunger length at settings of 0.0 (lowest position), 3.5, 7.0, and 10.0 mm (highest position). The shortest plunger length at which the participant bore weight through the pin and engaged the springs (Figure 1F) was identified.

### 2.4. Out-of-lab take-home testing

During the out-of-lab take-home test, the more compact design of the spring pin system was used (Figure 1B), and both pin sensor data and limb-socket distance sensor data were collected. Before the participant arrived, the liner to be worn was inserted and pushed deep into the socket (no residual limb) to determine the minimum possible pin height. The minimum possible pin height was used later in data presentation to provide a meaningful reference for clinical interpretation.

After arriving at the lab, the participant donned the test socket. The panels were kept at the neutral position, i.e., panels flush with the surrounding socket, for the duration of the protocol. The participant walked on the treadmill at a self-selected walking speed for 4 min to achieve a stable pin height during stance phase. This baseline pin height and knowledge of the participant's usual pin notch were used to calculate an appropriate plunger length. At the appropriate length, the plunger contacted the pin 0.1 mm before the pin entered the participant's usual pin notch. Once the pin was pushed into that notch and the ratchet locked into place, the slight pre-stress in the springs (38.4 N/mm × 0.1 mm = 3.84 N) ensured that at distal weight bearing forces greater than 3.84 N the pin provided a consistent rate of resistance (N/mm deflection) against the bottom of the residual limb.

The proper plunger length was installed in the spring pin system. The participant walked on the treadmill a second time to evaluate if the pin height data were within the sensor's measurement range (Corrections were not needed during any participant tests). The participant walked on streets and paved paths outside the lab for approximately 30 min to evaluate if further adjustment to the prosthesis was needed (not needed for any participant). Participants left the lab and wore the investigational prosthesis in their free-living environment for approximately one week (5–7 days). Data were collected to a portable data logger, similar to that described in prior work (12).

### 2.5. Data analysis

Pin sensor data collected from the in-lab and out-of-lab sessions were converted from raw signal counts to mm using the calibration data. To convert it to force (in N), the pin height data was zeroed to the pin height where the springs began to compress then multiplied by the spring constant to calculate force.

The out-of-lab data were segmented into prosthesis days using a strategy similar to that used in our prior work (12). A prosthesis day was defined as the first don longer than 30 min. If a don shorter than 30 min occurred less than 60 min before the start of the first don, then that don was considered the beginning of the prosthesis day. The end of the prosthesis day was defined as the start of a doff period longer than 60 min such that there were no donned periods longer than 30 min between this point and the start of the beginning of the next prosthesis day. Data within prosthesis days were classified as walks, shifts, stationary, partial doff, or full doff, using methods from prior work, summarized in Supplementary Figure F1. We calculated the time spent at each activity and expressed it as a percentage of the sum of all prosthesis day durations. Walks were further classified as bouts (≥5 steps) or low locomotion (<5 steps), and shifts were further classified as stand shifts or sit shifts. For both the in-lab and out-of-lab sessions, further analysis was carried out only on steps within walking bouts. The minimum pin height during stance phase and the maximum pin height during swing phase for each step were determined. They were termed the *stance phase minimum* and *swing phase maximum*, respectively. Means of the stance phase minima and swing phase maxima for each 2-min walk from the in-lab study and for all steps within bouts for the out-of-lab study were calculated. The surface area of the relatively stiff part of the umbrella on the bottom of the liner was calculated and used to approximate the pressure applied to the bottom of the residual limb. This calculation, termed the *tissue pressure* (in kPa), assumed that all force applied through the umbrella to the springs was evenly distributed over the bottom of the participant's residual limb.

For the out-of-lab sessions, data from the distance sensors embedded in the socket wall were converted from counts to mm using calibration data, and that data was thermally compensated to account for variations in socket temperature as described in prior work and summarized in Supplementary Presentation S3. For all steps within walking bouts, stance phase minima from the pin sensor were plotted against socket sensor stance phase minima to investigate relationships between the variables.

## 3. Results

### 3.1. Participants

Three participants executed the in-lab protocol (#1, #2, #3), and three executed the out-of-lab protocol (#1, #2, #4). All participants had their amputation as a result of trauma, were at a K-3 or K-4 level of activity, and traditionally wore a locking pin suspension system with a dynamic response foot. Participants were 36–76 years in age, and their amputee-adjusted body mass index (BMI) was between 24.7 and 34.7 kg/m^2^. Participant and prosthesis characteristics are summarized in Table 1. The umbrella diameter for the investigational prosthesis was the same size as the participant's traditional liner for participant #1 (93.4 mm), larger for participant #2 (74.7 mm vs. 72.4 mm), and smaller for participant #3 (68.4 mm vs. 71.8 mm) and participant #4 (74.7 mm vs. 86.3 mm). The relatively stiff part of the umbrella was of diameter 70.0 mm for participant #1, 60.0 mm for participants #2 and #4, and 68.4 mm for participant #3.

**Table 1:** Participant characteristics.

Partic	Gender	Age (year)	BMI (kg/m^2^)	Time since Amp (year)	Smoker	*K*-Level	Limb shape
1	M	59	33.0	32	Never	4	Cylindrical, short, fleshy
2	M	62	24.7	38	Never	4	Conical, medium, bony
3	M	36	34.7	16	Yes	4	Cylindrical, medium, muscular
4	M	76	27.3	48	Yes, 46 years ago	3	Conical, short, bony

### 3.2. Bench tests

**Figure 4:**
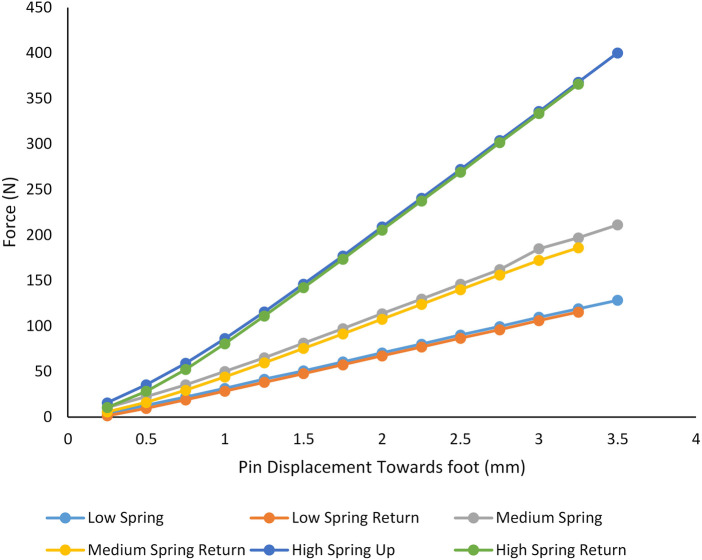
Bench test results. A testing machine was used to compression test the three different sets of springs. Stiffnesses of 34.3, 62.7, and 121.4 N/mm were measured. The lowest stiffness set of springs (red and blue lines) was used for all participants.

The force-displacement data from mechanical testing of the spring assembly showed a linear response with minimal hysteresis (Figure 4). The spring constants for the in-lab system (Figure 1A) were 34.3, 62.7, and 121.4 N/mm for the three sets of springs tested. During the in-lab evaluations, the lowest stiffness springs were deemed the most appropriate for all three participants, achieving sufficient sensitivity to detect a meaningful signal while at the same time not bottoming out or causing participant discomfort. In the out-of-lab tests where the more compact system configuration was implemented (Figure 1B), the spring constant for all participants was 38.7 N/mm.

### 3.3. In-lab tests

**Figure 5:**
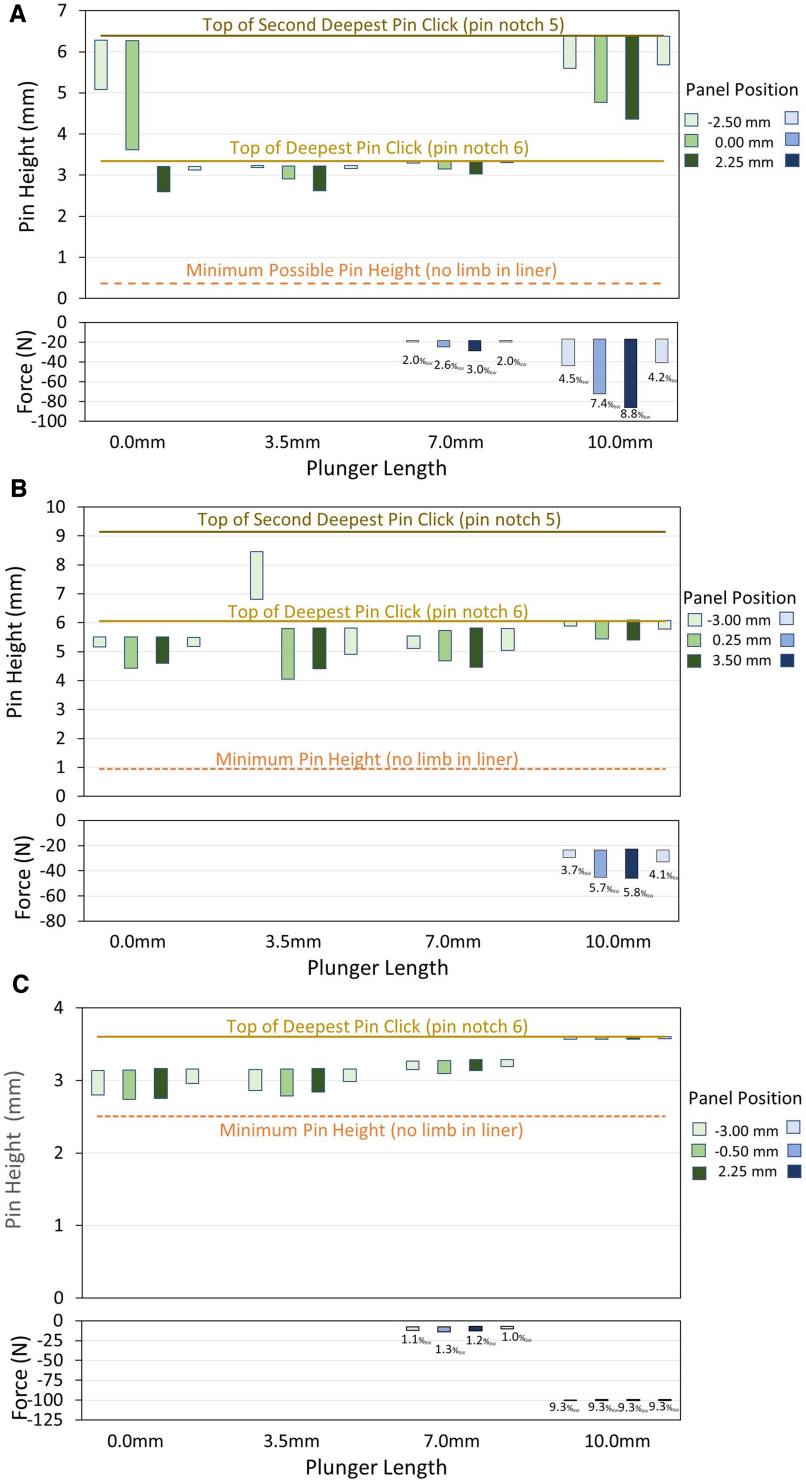
(**A**–**C**) in-lab test results. Data for participant #1 (**A**), #2 (**B**), and #3 (**C**) are shown. The upper plots are pin height in mm, and the lower plots are distal weight bearing in *N*. In the pin height plots, the bar plots span the range from the mean minimum to the mean maximum pin height for all steps within the 2-min walk. The transitions between pin notches are shown as solid yellow horizontal lines. The minimum possible pin height (dashed orange horizontal line) is the maximum depth of the liner umbrella in the socket without the residual limb in the liner. In the distal weight bearing plots, the mean percent body weight (% BW) during stance phase is printed underneath the bar for each 2-min walk. Once the plunger length was high enough for the pin to contact the plunger and apply force to the springs during stance phase, participants showed a decrease in pin height and an increase in percent weight bearing when the panels were loosened from the tightest to both the midpoint and loosest positions, and they showed an increase in pin height and a decrease in percent weight bearing when the panels were tightened from the loosest to the tightest position.

While wearing the test prosthesis at the 0.0 mm plunger length, no participant reached the deepest possible pin height (indicated with a dashed orange line in Figures 5A–C). There was an air gap of between 0.3 mm and 3.2 mm between the umbrella and the bottom ledge of the socket (Figure 2B) at the 0.0 mm plunger length during stance phase for the in-lab tests.

For all three participants, at least one plunger length produced distal weight bearing (7.5 and 10.0 mm for participants #1 and #3; 10.0 mm for participant #2) (Figures 5A–C). The highest mean distal weight bearing during stance phase of a 2-min walk at the highest plunger length was 8.8% BW for participant #1, 5.8% BW for participant #2, and 9.3% BW for participant #3.

At plunger lengths that produced distal weight bearing, all three participants showed a decrease in pin height and an increase in percent weight bearing when the panels were loosened from the tightest to both the midpoint and loosest positions (Figures 5A–C). Participants showed an increase in pin height and a decrease in percent weight bearing when the panels were tightened from the loosest to the tightest position. Participant #1 made an unsolicited comment during the in-lab tests that his socket was more comfortable when he was distal weight bearing.

At plunger lengths that produced distal weight bearing (7.0 and/or 10.0 mm depending on the participant), the coefficient of variation in pin height (standard deviation divided by the mean) during walking was higher for the stance phase minima than the swing phase maxima for each participant. The maximum coefficient of variation in the stance phase minima at a socket panel position was 2.8%, 2.4%, and 1.0% for participants #1, #2, and #3, respectively, and those for the swing phase maxima were <0.2% for all three participants.

**Figure 6:**
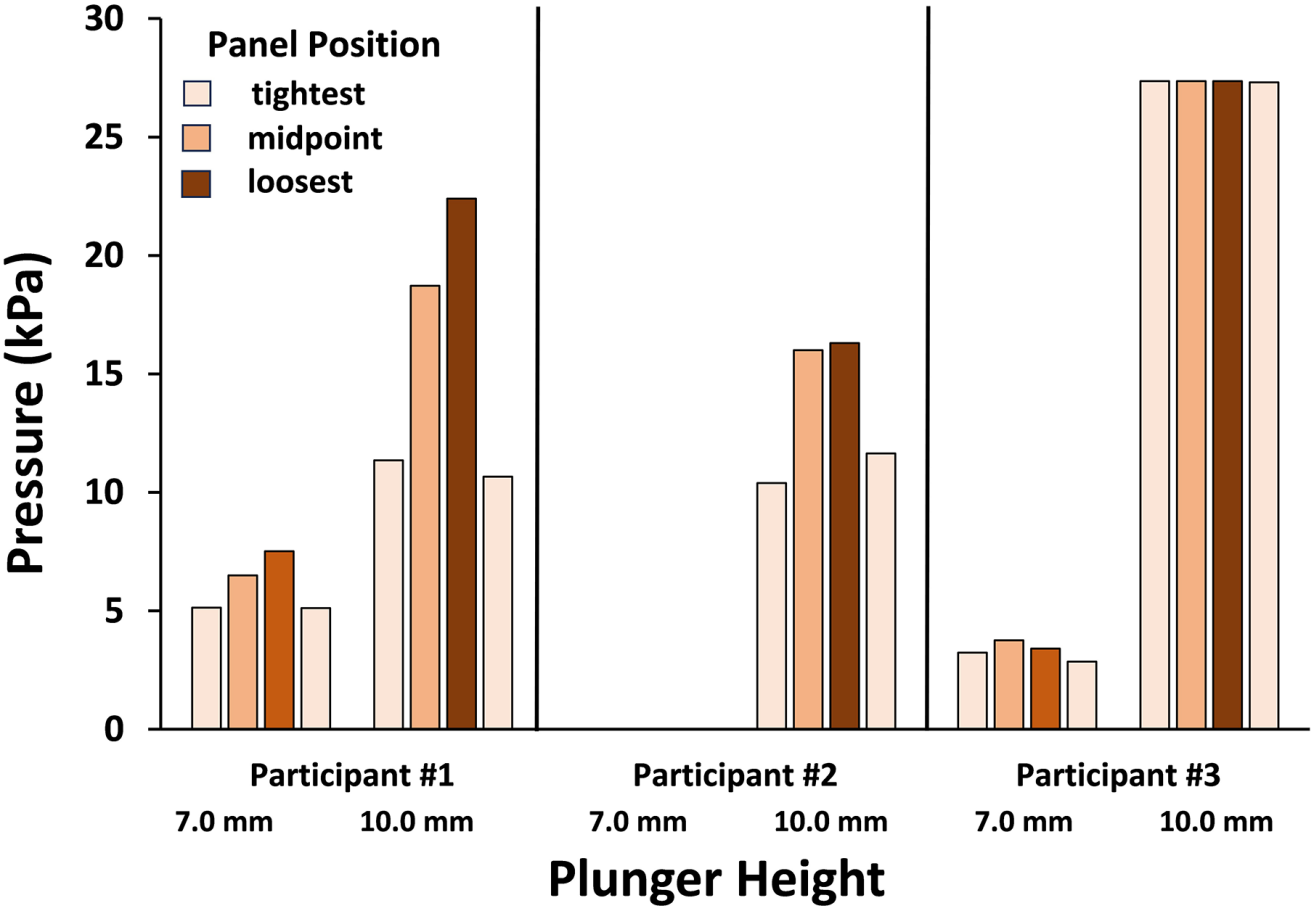
Pressures on the distal residual limb during the in-lab test. It is assumed that the applied force was evenly distributed over the umbrella surface. Data for the 7.0 and 10.0 mm plunger lengths are shown for the three participants. Participant #3 at the 10.0 mm plunger length demonstrated relatively consistent pressure compared to participants #1 and #2 presumably because the difference between the swing phase maximum and stance phase minimum was low, and the force applied to the shuttle lock to hold the pin at the deepest notch was relatively high.

For the trials where the springs were compressed, the calculated tissue pressures on the bottom of the residual limb during stance phase ranged up to 22.4 kPa for participant #1, 16.3 kPa for participant #2, and 27.4 kPa for participant #3 (Figure 6). Distal pressure increased as the panels were loosened for participant #1 and #2, while participant #3 had relatively consistent distal pressure across different panel positions.

Participants #1 and #2 did not reach their deepest pin notch in some of the 2-min walks. Participant #1 reached only the second deepest pin notch (notch 5) during the first two walks at the 0.0 mm plunger length but not the other two walks, and during all four walks at the 10.0 mm plunger length. Participant #2 reached only the second deepest notch (notch 5) during the first walk at the 3.5 mm plunger length, but in all other walks he reached the deepest pin notch (notch 6).

### 3.4. Out-of-lab take-home tests

**Figure 7:**
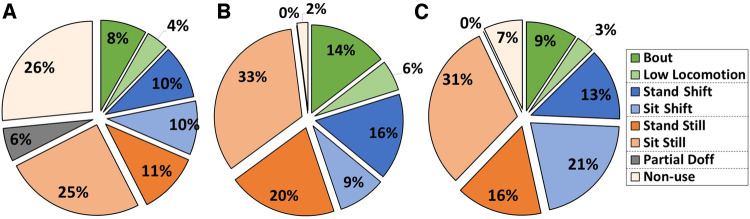
(**A**–**C**) participant activity during take-home testing. Data for participant #1 (**A**), #2 (**B**), and #4 (**C**) are shown. Data are presented as a percentage of the sum of the prosthesis day duration. Participants spent 32%–46% of their prosthesis day duration conducting walks and shifts (blue and green sections) but varied considerably as to their distributions among the activities.

Participants #1, #2, and #4 spent 32%, 45%, and 46%, respectively, of their sum prosthesis day time conducting walks and shifts (Figures 7A–C). Participant #1 spent 32% of his time partially or fully doffed, while participants #2 and #4 spent only 2% and 7%, respectively, of their time partially or fully doffed.

The mean stance phase minimum was 2.5 mm for participant #1, 9.4 mm for participant #2, and 2.4 mm for participant #3 (Table 2). Participant #2's pin height was higher during the out-of-lab test compared with the in-lab test; he was at pin notch 4 instead of notch 6 during the out-of-lab test. The air gap during stance phase during the out-of-lab tests was 1.5–3.1 mm for participant #1; 7.3–9.7 mm for participant #2; and 1.0–3.2 mm for participant #4. The coefficient of variation (standard deviation divided by the mean) for stance phase minimum pin height was 10.4% for participant #1, 0.7% for participant #2, and 16.9% for participant #4, while the coefficient of variation for swing phase maximum pin height was less, 2.3%, 0.4%, and 3.3% for participants #1, #2, and #4, respectively.

**Table 2:** Out-of-lab data.

Partic.	Variable	Day 1	Day 2	Day 3	Day 4	Day 5	Day 6	Day 7	Day 8	Day 9	Day 10	All steps
#1	Min depth (mm)	2.3 (0.3)	2.4 (0.2)	2.4 (0.2)	2.6 (0.2)	2.8 (0.1)						2.5 (0.3)
	Max force (*N*)	−31.3 (10.5)	−30.6 (8.8)	−30.4 (7.9)	−21.5 (8.0)	−13.2 (4.6)						−26.9 (9.9)
	Max DWB (%BW)	3.2 (1.1)	3.1 (0.9)	3.1 (0.8)	2.2 (0.8)	1.4 (0.5)						2.7 (1.0)
	Max tiss Pr (kPa)	8.1 (2.7)	8.0 (2.3)	7.9 (2.1)	5.6 (2.1)	3.4 (1.2)						7.0 (2.6)
#2	Min depth (mm)	9.5 (0.03)	9.4 (0.08)	9.4 (0.05)	9.4 (0.05)	9.5 (0.03)	9.5 (0.03)	9.5 (0.03)	9.5 (0.02)	9.4 (0.04)	9.5 (0.05)	9.4 (0.06)
	Max force (*N*)	−9.0 (1.2)	−12.0 (3.3)	−11.0 (1.8)	−10.3 (1.8)	−8.9 (1.2)	−8.4 (1.0)	−8.6 (1.3)	−8.9 (1.0)	−10.0 (1.4)	−9.1 (1.9)	−10.1 (2.4)
	Max DWB (%BW)	1.1 (0.1)	1.5 (0.4)	1.4 (0.2)	1.3 (0.2)	1.1 (0.2)	1.1 (0.1)	1.1 (0.2)	1.1 (0.1)	1.3 (0.2)	1.1 (0.2)	1.2 (0.3)
	Max tiss Pr (kPa)	3.2 (0.4)	4.3 (1.2)	3.9 (0.6)	3.6 (0.6)	3.2 (0.4)	3.0 (0.4)	3.1 (0.5)	3.2 (0.3)	3.5 (0.5)	3.2 (0.7)	3.6 (0.9)
#4	Min depth (mm)	1.9 (0.3)	2.4 (0.4)	2.4 (0.4)	2.6 (0.3)	2.2 (0.2)	2.5 (0.4)	2.3 (0.3)				2.4 (0.4)
	Max force (*N*)	−55.6 (12.6)	−35.9 (15.4)	−34.2 (14.4)	−28.6 (10.8)	−42.5 (9.2)	−30.5 (14.3)	−36.5 (11.7)				−36.1 (15.5)
	Max DWB (%BW)	6.4 (1.5)	4.2 (1.8)	4.0 (1.7)	3.3 (1.2)	4.9 (1.1)	3.5 (1.7)	4.2 (1.4)				4.2 (1.8)
	Max tiss Pr (kPa)	19.7 (4.5)	12.7 (5.4)	12.1 (5.1)	10.1 (3.8)	15.0 (3.3)	10.8 (5.1)	12.9 (4.1)				12.8 (5.4)

Mean (SD) stance phase results for each day and for all steps.

**Figure 8:**
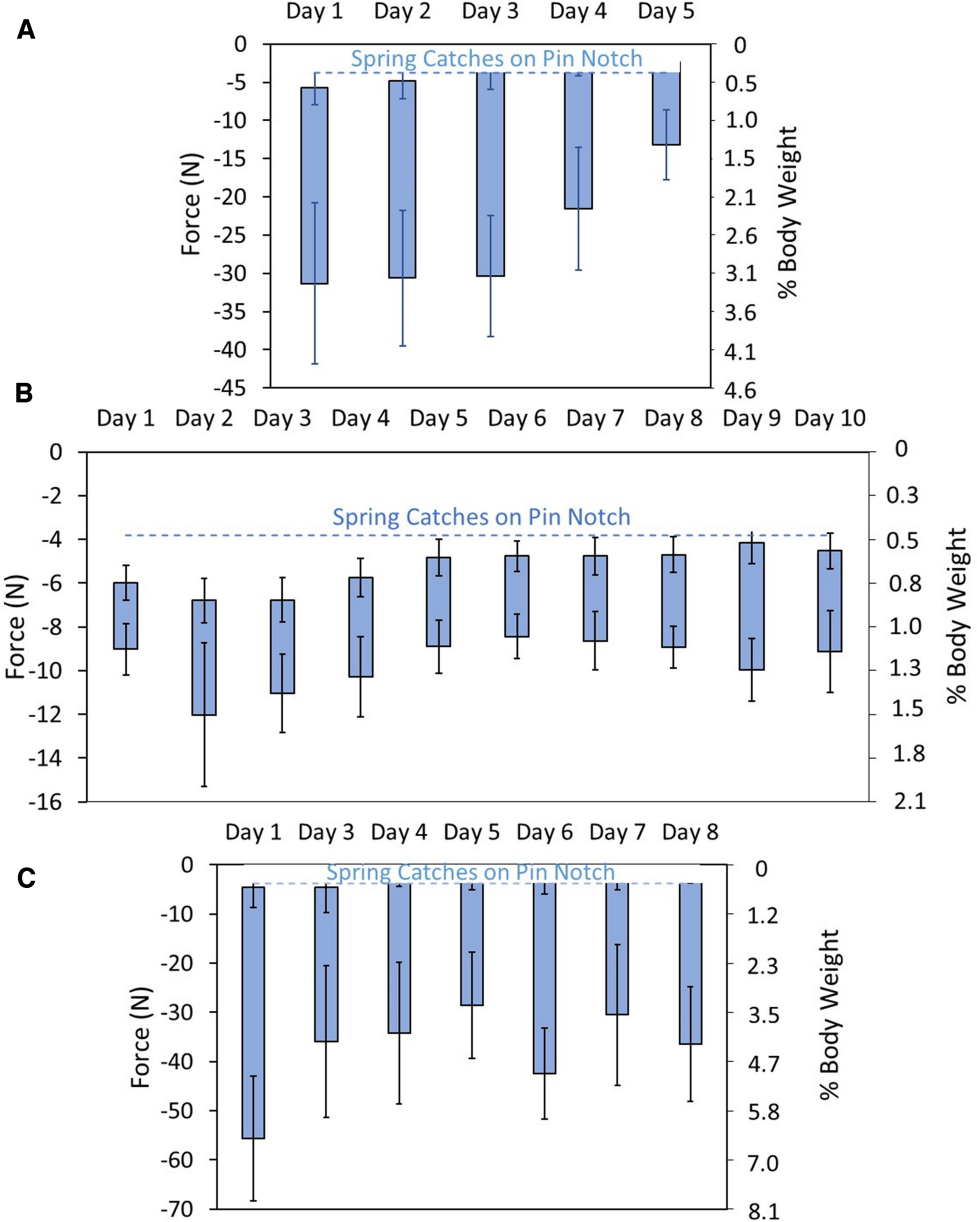
(**A**–**C**) distal weight bearing during take-home testing. Data are shown in units of force (*N*) (left axis) and % BW (right axis). The dashed blue line is the pin height at which the pin transitioned to the participants’ deepest notch. The coefficient of variation for stance phase percent distal weight bearing and tissue pressure was 37% for participants #1 (**A**), 25% for participant #2 (**B**), and 43% for participant #4 (**C**).

For all participants during the out-of-lab tests, the mean stance phase weight bearing force delivered to the bottom of the residual limb was under 5.0% BW (range 1.1% to 6.4%), and the mean of the maximum tissue pressure delivered was 12.8 kPa or less (right column, Table 2). The coefficient of variation in distal weight bearing and tissue pressure was higher than that for pin height because the zero reference for the pin height was at the top of the pin lock while that for weight bearing and tissue pressure was the pin height at which the springs began to compress. The coefficient of variation for stance phase percent distal weight bearing and tissue pressure was 37% for participants #1, 25% for participant #2, and 43% for participant #4. In many of the steps during swing phase for participants #1 and #4, the pin was at the top of the displacement range, i.e., the notch was against the ratchet, which restricted further proximal motion of the pin (dashed blue line in Figures 8A–C).

**Figure 9:**
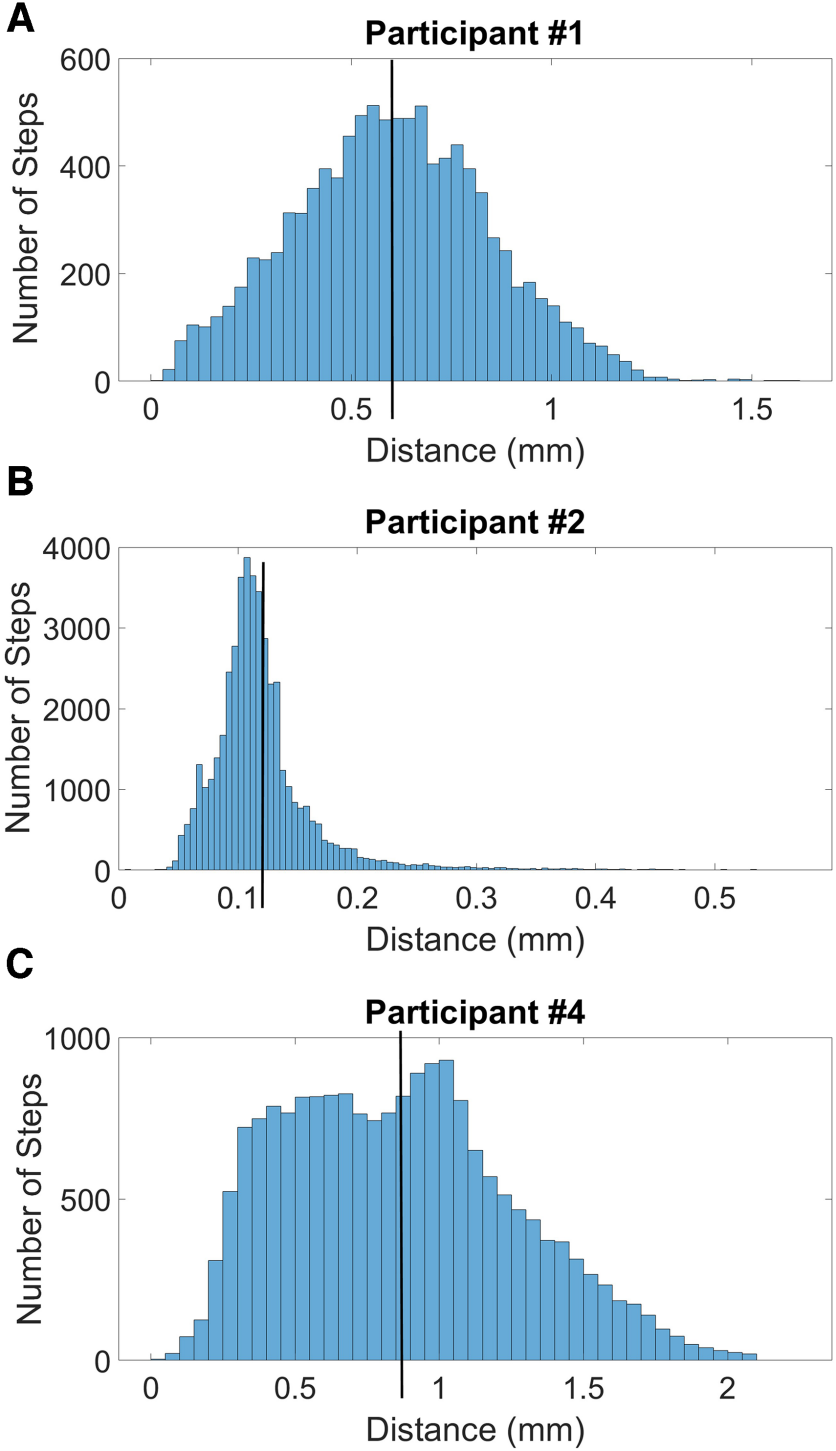
(**A**–**C**) histograms of swing phase maximum minus stance phase minimum during all steps during take-home testing. The black vertical lines indicate the mean pistoning magnitudes for all steps. The histogram for participant #1 (**A**) is relatively normally distributed, participant #2 (**B**) is right-skewed, and participant #4 (**C**) is bimodal.

Pin pistoning (maximum—minimum pin height) during all walking bout steps was a mean of 0.6 mm for participant #1, 0.1 mm for participant #2, and 0.8 mm for participant #4. The histograms of pistoning distance for all days combined were normally distributed for participant #1, right skewed for participant #2, and bimodal for participant #4 (Figures 9A–C). Histogram data separated by day were relatively normally distributed for participant #1, right-skewed for participant #2, and bimodal on 4 of the 7 days for participant #4 (Supplementary Figure F2), consistent with the all walking steps plots in Figures 9A–C.

Pin height plotted against sensed distance for the six sensors positioned in the socket wall did not show strong linear relationships (Supplementary Figure F3). Higher pin heights tended to show higher sensed distances for some of the days and locations (e.g., Supplementary Figure F3A, anterior distal) but on other days and locations the plots were relatively flat (e.g., Supplementary Figure F3C, posterior mid-limb lateral). The upper edge of the pin notch was visible in data from some days (e.g., as a boundary at 9.6 mm pin height in Supplementary Figure F3B). Many days showed clusters of data (e.g., Supplementary Figure F3A, posterior mid-limb medial), indicating groups of steps at different sensed distances or at different pin heights. The range of pin heights across a day was lowest for participant #2 and highest for participants #1 and #4. Participant #4 noted that he felt less pistoning than normal during the take-home part of the out-of-lab test.

## 4. Discussion

The developed instrument has the potential to help researchers and practitioners better understand how prosthesis design variables and participant characteristics affect limb motion and distal weight bearing in people using locking pin suspension. Monitored data may prove useful in clinical care. Potentially, the signal could be part of a feedback control system for an auto-adjusting socket to maintain a certain distal weight bearing level that improved outcome. Results from the present study on a small group of participants demonstrated that the instrumentation measured distal weight bearing with good sensitivity and the magnitude of distal weight bearing could be controlled via adjustment of the plunger length in the mechanism that supported the locking pin.

Changes in limb fluid volume during the in-lab protocols may explain why participants #1 and #2 were at different pin notch settings during some of the 2-min walks at the 0.0 mm and 3.5 mm plunger lengths (Figures 5A,B). Studies on people with transtibial amputation have typically shown a gradual decrease in limb fluid volume during ambulation right after donning (13). The range in pin height [swing phase maximum minus stance phase minimum (length of the green bars in Figures 5A–C)], decreased when participants transitioned to the next deeper serration in the ratchet (pin notch). Once participants transitioned to a deeper notch, the top of their range was lower in the socket, restricting pin proximal displacement during swing phase.

Results from the in-lab tests (Figures 5A–C) demonstrated that adjusting the plunger length had the expected effect on the pin height and distal weight bearing measurements. At high plunger lengths (7.0 and 10.0 mm for participants 1 and 3; 10.0 mm for participant #2) the springs compressed, and load bearing on the distal end of the residual limb occurred. For each participant, the pin positions for the 7.0 mm plunger length were higher than those for the 3.5 mm plunger length, and the pin positions for the 10.0 mm plunger length were higher than those for the 7.0 mm plunger length. Participant #1 was pushed up a pin notch (to pin notch 5) at the 10.0 mm plunger length. The force applied through the springs to the bottom of the residual limb at the 10.0 mm plunger length varied considerably across participants, in part reflecting their BMI. The force was 41.0–86.2 N (4.2–8.9% BW) for participant #1 (33.0 kg/m^2^ BMI), 29.4–46.1 N (3.0–4.7% BW) for participant #2 (24.7 kg/m^2^), and 100.3–100.5 N (10.3–10.3% BW) for participant #3 (34.7 kg/m^2^). Participant #3's range was less than the other two participants, presumably because his stance phase minima was just under the pin position transition to the next serration in the rastered pin (notch 6) (Figure 5C). The compressive force applied to the pin to transition to notch 6 and for the shuttle lock to hold it there, was relatively high. The umbrella for participant #3's investigational prosthesis, unlike that for participant #1 and #2, was slightly smaller than the umbrella in his traditional socket, which may have contributed to his elevated distal weight bearing.

If a researcher or prosthetist sought to use this system to monitor distal weight bearing with minimal disruption compared to the participants' regular socket, then setting the start of the spring resistive force just above the lowest pin notch the user experiences during regular use may be appropriate, as performed in the out-of-lab tests in this study. Using a lower spring stiffness would further reduce the applied distal weight bearing force though with an increased risk of bottoming out the springs in the mechanism. If it were of interest to increase distal weight bearing to investigate its potential benefit towards participant well-being, enhancing blood flow via a pumping effect on distal limb tissue for example (1), then the spring resistive force should be started at a high plunger length, essentially shortening the socket, or spring stiffness should be increased.

The day-to-day differences in stance phase distal weight bearing measured in the take-home part of the out-of-lab study were well within the range of the sensor's measurement capabilities. The day-to-day fluctuation in pin position could have been due to day-to-day changes in several variables including limb fluid volume, sock thickness, the type of activity conducted, or some other variable. All distal weight bearing forces measured in the out-of-lab study (daily means ranged from 1.1 to 6.4% BW) and their standard deviations were relatively low (Table 2). Because they were so much less than the threshold pain tolerance for people with transtibial amputation reported in the literature [13%–48% of maximum vertical ground reaction force for Katz et al. (3); 17.2% BW (SD 13.1) for Persson and Liedberg (4)], it is unlikely participants would find the system at these plunger lengths painful or disruptive. No complaints were reported during take-home use. Participant #4, however, did report that he experienced less pistoning than with his normal prosthesis during take-home use which he found favorable. This may have been because the distal contact helped reduce motion of the limb in the socket in the sagittal plane. It is also possible that low level distal weight bearing improved proprioception. These conclusions are conjecture and would need to be tested through rigorous scientific investigation.

When we calculated distal limb pressure by assuming the distal limb force was evenly distributed over the relatively stiff bottom part of the participant's liner umbrella, mean tissue pressures were up to 27.4 kPa for the in-lab study and up to 12.8 kPa for the out-of-lab study. The reason that pressures were higher for in-lab than out-of-lab is that the plunger lengths were higher (trials 3 and 4), essentially shortening the length of the socket more than for the out-of-lab test. In circulatory studies conducted in 12 able-bodied participants (no co-morbidities) over the anterior lateral proximal aspect of the tibia, Sangeorzan et al. (14) found that a mean pressure of 9.5 kPa was sufficient to occlude blood flow in the skin. It is thus possible that blood flow was occluded during the stance phase of some steps in the take-home participants. However, pressures that intermittently occlude blood flow (e.g., during stance phase within a step) may be acceptable and even favorable provided the blood vessels re-open and the tissue re-perfuses during swing phase, and reactive hyperemia is not induced. This interpretation is conjecture and the clinical impact of intentionally increasing intermittent distal weight bearing would need to be thoroughly studied before it is implemented in clinical care.

The coefficient of variation in stance phase minima in the walking bouts during the out-of-lab tests (10.4%, 0.7%, and 16.9%, respectively) was considerably greater than that in the 2-min bouts during the in-lab tests (<3.0% for all participants). This difference likely reflects changes in walking style, speed, terrain, direction of ambulation, and bout duration in participants' take-home environments compared with the treadmill walking in the lab. Changes in limb fluid volume may also have contributed. Repeatability tests measuring pylon force data in the laboratory have shown considerable variability, leading Zahedi et al. to suggest that field-collected data variability should be evaluated and that information considered in clinical decision making (15). Similar to Zahedi et al. (15), we conclude that pin height and distal weight bearing data may be useful in clinical care. Investigations are needed to quantify and rank the sources of within-day and between-day variability in field-collected force data (16). Achieving that understanding would facilitate determining how to use field-collected data to improve patient outcome.

To be capable of distal weight bearing adjustment during clinical use, the mechanical design of the device would need to be advanced, e.g., an adjustable knob placed on the outside of the device or a wireless device such as a fob or phone app. Potentially, an automated system could be created to adjust the plunger length based on the distal weight bearing measurement, keeping it within a certain percentage body weight range specified by the prosthetist.

At the time of Katz et al.'s (3) and Persson and Liedberg's (4) studies, before elastomeric liners and locking pin suspension were commonly used in clinical practice, practitioners considered some distal weight bearing necessary to reduce edema in the distal residual limb (1). The cyclic distal loading during walking was intended to have a pumping effect on the distal limb, driving out edematous fluid. Elastomeric liners, however, which are nowadays commonly used in clinical practice, introduce an additional strategy to help limit edema in distal limb tissues—radial compressive stress is applied via the elastic properties of the liner polymer. Implementation of edema management using elastomeric liners may have discouraged practitioners from designing sockets to meaningfully cyclically load the distal end of the residual limb. However, in clinical practice sockets are designed to achieve at least some distal weight bearing. It is unknown if no distal weight bearing using locking-pin elastomeric liners, is less favorable to residual limb tissue health than some cyclic distal weight bearing, i.e., cyclic pumping, during ambulation. It is also unknown to what degree no distal weight bearing occurs in clinical practice.

The air gap, the vertical distance between the umbrella and the ledge near the bottom of the socket (Figure 2B), may allow the distal end of the residual limb to translate anterior-posterior, particularly if proximal residual limb enlargement is the source of the air gap. During the present investigation, two of the four participants made unsolicited comments that the socket was more comfortable with distal weight bearing than without it. It is possible that load applied to the bottom of the residual limb reduced distal limb sagittal plane motion and thus reduced local stresses over the anterior distal tibia. In other words, contact with the bottom of the socket may have stabilized the distal residual limb. It is also possible that some cyclic distal weight bearing facilitated blood flow in distal residual limb tissues, improving tissue oxygenation, proprioception, and participant comfort. These possibilities are conjectures and would need to be tested through rigorous scientific investigation.

A next step to bring this technology closer to everyday clinical use is to develop a clinical fitting procedure to determine target values and ranges for distal weight bearing. In preliminary efforts conducted since completing this study, we found that participants with protective sensation were able to discern a 0.50-mm change in plunger length, corresponding to a 19.2 N change in force and a 2.7% BW change for a 72 kg (160 lb) participant. Thus, we propose that 0.50-mm increments, using 38.7 N/mm stiffness springs, should be used in initial studies to develop clinical fitting procedures.

There was not a strong linear relationship between sensed liner-socket distance and pin height (Supplementary Figure F3), suggesting that variables other than limb vertical position affected their relationship. Variables that may have changed during the day and interacted with each other include residual limb volume, thickness of socks worn, activities conducted, tissue mechanical property changes, and friction between the limb and liner or between the liner and socket, to name a few. It would be interesting to determine what caused some of the data to cluster at different sensed distances and pin heights during take-home testing (Supplementary Figure F3).

A limitation of the spring pin system used in this investigation is that the weight of the system may have affected how participants used the test prosthesis. The added weight (326.4 g for the in-lab system (Figure 1A), and 241.0 g for the out-of-lab system (Figure 1B) was comparable to an electronic elevated vacuum system. The short-term nature of the investigation and the limited number of participants tested limit generalized application of the study results, but they do provide a base from which to design larger clinical studies.

## 5. Conclusion

The developed instrument had sufficient resolution to pick up detailed information about distal weight bearing in the present investigation, for example detecting changes in distal weight bearing when socket size was adjusted as well as sensing day-to-day fluctuations. The degree of distal weight bearing was adjustable, and some participants preferred greater distal weight bearing than that in their traditional socket. Future studies should investigate sources of variability in take-home test data and its relevance to patient outcomes. A clinical fitting procedure to establish target values and ranges should be pursued.

## Data Availability

The original contributions presented in the study are included in the article/**Supplementary Material**, further inquiries can be directed to the corresponding author.
