# Supplementary material for: Distal weight bearing in transtibial prosthesis users wearing pin suspension
Source: Front Rehabil Sci. 2023 Dec 21;4:1322202. doi: 10.3389/fresc.2023.1322202 (PMC10773776; doi:10.3389/fresc.2023.1322202)

Supplementary Presentation P1. Pin Sensor Calibration.

To calibrate the pin sensor in a participant's socket, the following procedure was used. The pin plunger (see Fig. 1A,B in the text) was retracted downward so that the pin could not contact the plunger during the procedure. The pin was screwed into a 1 kg weighted assembly (in *A* below). The bottom of the 3D printed part supporting the 1 kg weight in *A* was designed to fit into the recess on the top surface of the locking mechanism (shuttle lock) in the socket, which ensured that the pin and weight were centered along the longitudinal axis of the locking mechanism. This configuration was the deepest possible position of the locking pin in the socket. Next, 22 printed spacers (top left in *A*) were placed on the locking pin one at a time and the weight assembly placed in the socket (*B* below), creating a pin height ranging from 0.00 to 16.02 mm. Data from the pin sensor were collected at each height. A polynomial was fit to the plot of pin depth and sensor counts (an example plot is shown in *C* below). This calibration curve was used to process participant pin sensor data, converting it to height in mm where the 0.00 mm was most distal possible pin position.

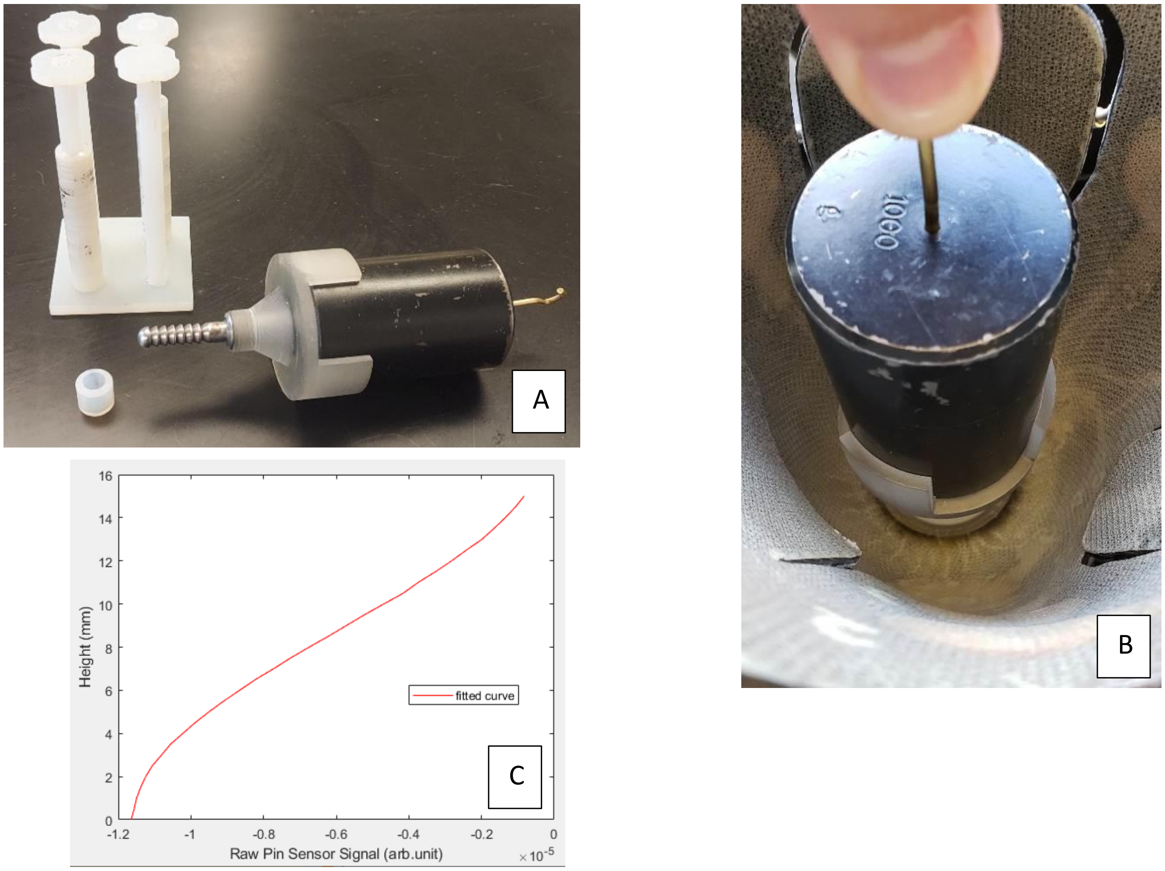

Supplement: Supplementary file 2 [file Presentation1.pdf]
