# Supplementary material for: Distal weight bearing in transtibial prosthesis users wearing pin suspension
Source: Front Rehabil Sci. 2023 Dec 21;4:1322202. doi: 10.3389/fresc.2023.1322202 (PMC10773776; doi:10.3389/fresc.2023.1322202)
Supplement: Supplementary file 3 [file Presentation2.pdf]

Supplementary Presentation P2. Sensor Locations.

Sensor locations were established using techniques described in detail in Ballesteros [2023]. Briefly, fiducial marks were identified at the following locations: an anterior midline defined by the tibial crest; a posterior midline defined midway between the hamstrings; the edge of the umbrella when the liner was fully seated into the socket; and the posterior trimline.

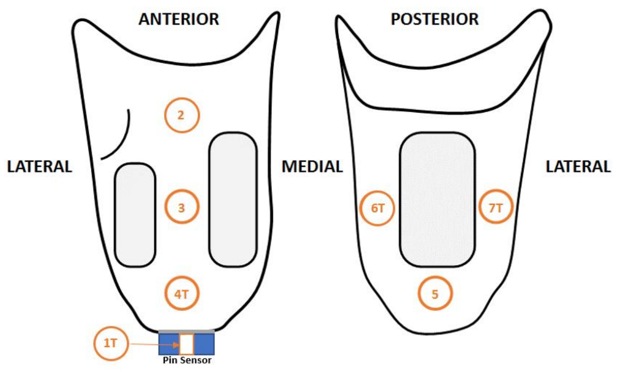

The six sensor locations on the socket are shown. The pin sensor is positioned underneath the socket.

1T= pin sensor; 2= anterior proximal; 3= anterior midlimb; 4T= anterior distal; 5= posterior distal; 6T= posterior medial midlimb; 7T= posterior lateral midlimb

Ballesteros D, Carter RV, Allyn KJ, Lanahan CR, Krout AJ, Hafner BJ, et al. Fabricating sockets with distance sensors for monitoring prosthesis use and socket fit. J Prosthet Orthot. 2023:10.1097/JPO.0000000000000464
