# Supplementary material for: Distal weight bearing in transtibial prosthesis users wearing pin suspension
Source: Front Rehabil Sci. 2023 Dec 21;4:1322202. doi: 10.3389/fresc.2023.1322202 (PMC10773776; doi:10.3389/fresc.2023.1322202)
Supplement: Supplementary file 4 [file Presentation3.pdf]

Supplementary Presentation P3. Distance Sensor Calibration.

For the out-of-lab sessions, raw signal data from the distance sensors embedded in the socket wall were converted to distance measurements in millimeters using the following procedure.

First, the ferrous liner to be worn by the participant was calibrated in a bench test setup. Data was collected and stored to the data logger while a sensor antenna was positioned at multiple heights above the ferrous liner, starting from zero. The antenna was fastened to an arm that was attached to a high-quality digital height gauge to execute this test. A calibration curve of sensor output in counts and distance in mm was generated. Since the shape of this curve was repeatable at different locations on the liner, a mean curve from three test locations was used. This procedure established a base calibration curve. Second, a residual limb-shaped balloon made of silicone was placed inside the ferrous liner to be worn by the participant, and the liner was placed inside the participant's test socket. The pin lock was engaged, the balloon was inflated to 34.5 kPa, and sensor data was collected. This second procedure established the flush condition, i.e., zero distance position, of the liner against the socket, setting the *y*-intercept in the calibration equation.

In addition to distance calibration, a thermal calibration procedure was executed. While the data logger was collecting data from the sensors, the socket was put in an incubator and slowly heated over a 1-h period to 38.0°C. The incubator door was opened, and the test socket was allowed to cool back to room temperature, a process that took between 50 and 80 min. A least-squares fit to the sensor data plotted against the thermistor data was calculated. Using the thermistor data that was continuously collected during participant use, the sensor data was corrected for temperature changes in the test socket.
