# Supplementary material for: Distal weight bearing in transtibial prosthesis users wearing pin suspension
Source: Front Rehabil Sci. 2023 Dec 21;4:1322202. doi: 10.3389/fresc.2023.1322202 (PMC10773776; doi:10.3389/fresc.2023.1322202)
Supplement: Supplementary file 5 [file Image1.pdf]

Supplementary Figure F1. Data Processing Flowchart for Identification of Activities.

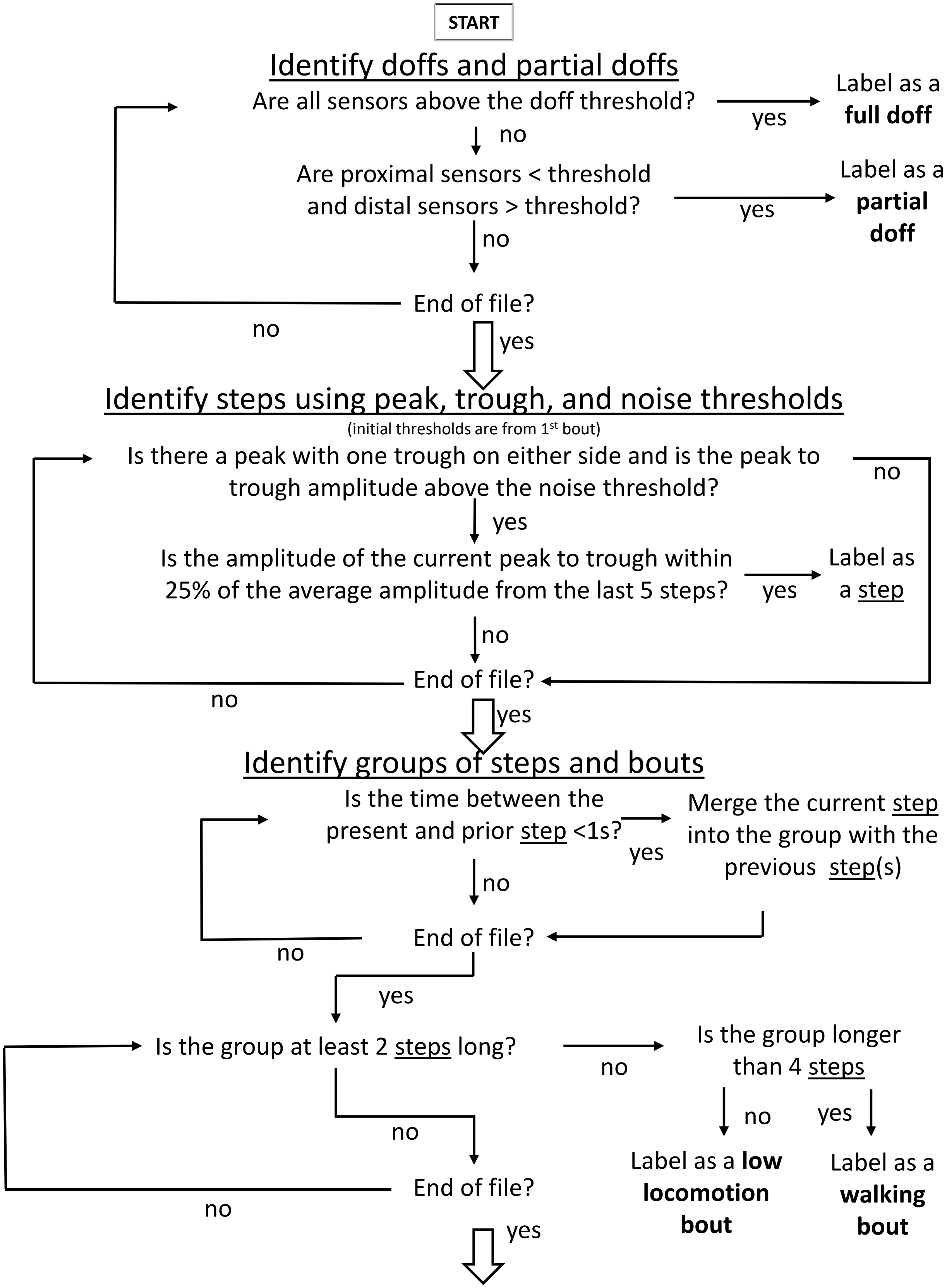

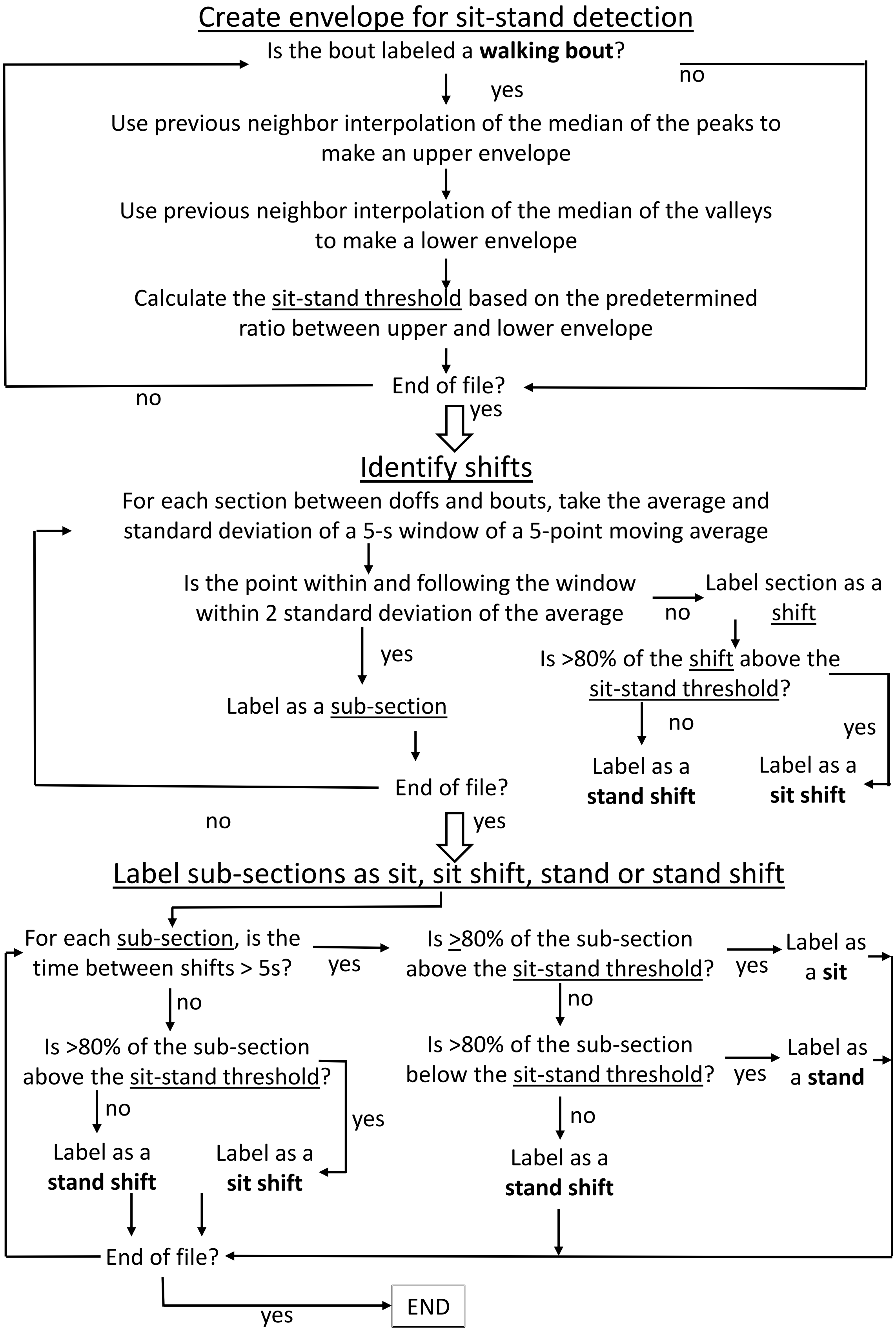
Create envelope for sit-stand detection
Is the bout labeled a **walking bout**?
yes
no
Use previous neighbor interpolation of the median of the peaks to make an upper envelope
Use previous neighbor interpolation of the median of the valleys to make a lower envelope
Calculate the sit-stand threshold based on the predetermined ratio between upper and lower envelope
End of file?
no
yes
Identify shifts
For each section between doffs and bouts, take the average and standard deviation of a 5-s window of a 5-point moving average
Is the point within and following the window within 2 standard deviation of the average
no
Label section as a shift
yes
Is >80% of the shift above the sit-stand threshold?
Label as a sub-section
no
yes
Label as a **stand shift**
Label as a **sit shift**
End of file?
no
yes
Label sub-sections as sit, sit shift, stand or stand shift
For each sub-section, is the time between shifts > 5s?
yes
Is ≥80% of the sub-section above the sit-stand threshold?
yes
Label as a **sit**
no
no
Is >80% of the sub-section above the sit-stand threshold?
Is >80% of the sub-section below the sit-stand threshold?
yes
Label as a **stand**
no
yes
no
Label as a **stand shift**
Label as a **sit shift**
Label as a **stand shift**
End of file?
yes
END
