# Supplementary figures and images for: Distal weight bearing in transtibial prosthesis users wearing pin suspension
Source: Front Rehabil Sci. 2023 Dec 21;4:1322202. doi: 10.3389/fresc.2023.1322202 (PMC10773776; doi:10.3389/fresc.2023.1322202)

Supplementary Figure F2. Pistoning Histograms.

Participant #1

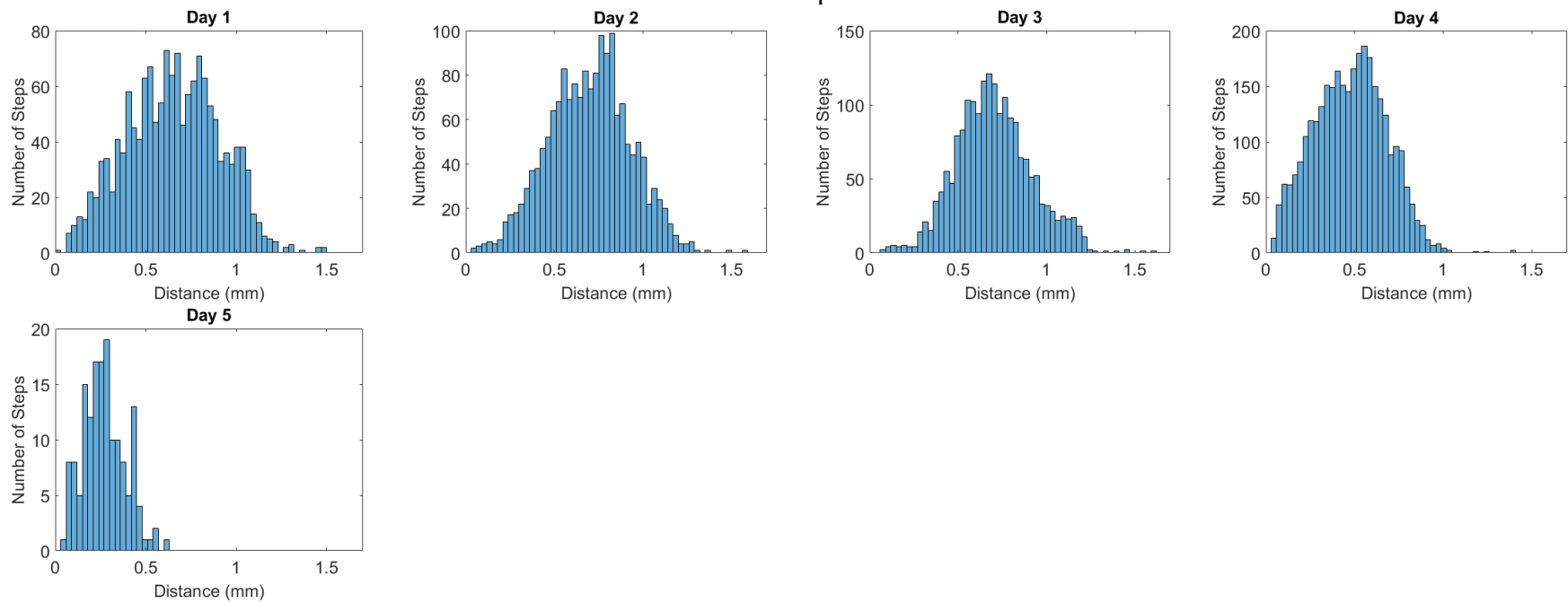

## Participant #2

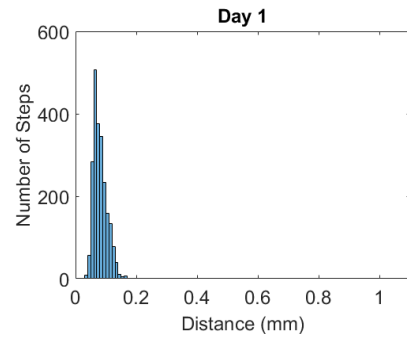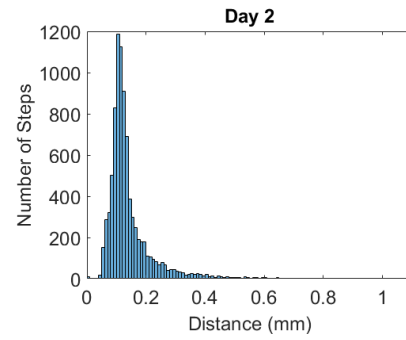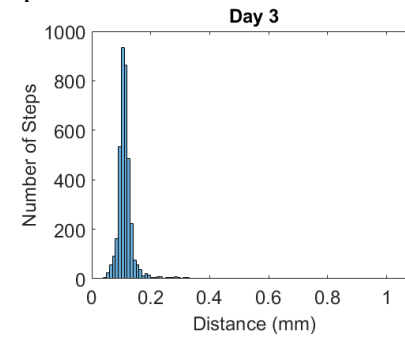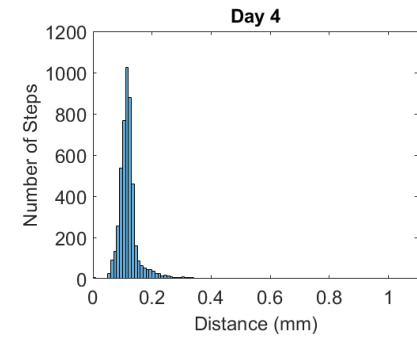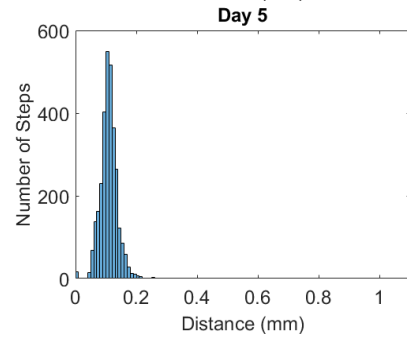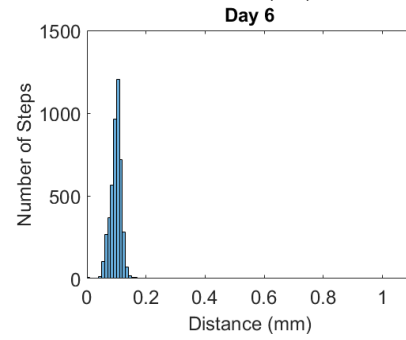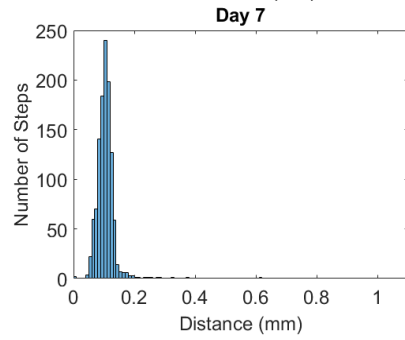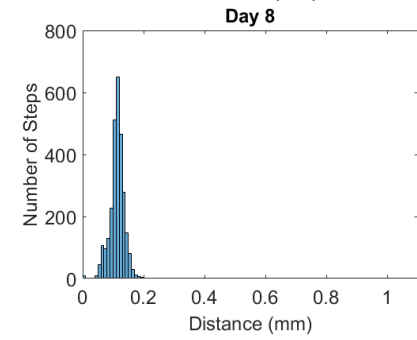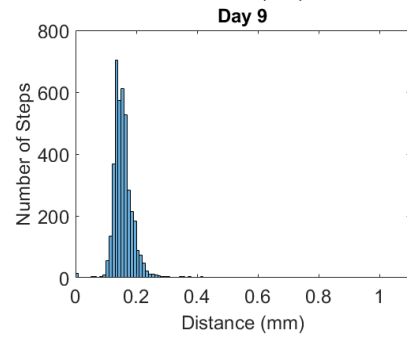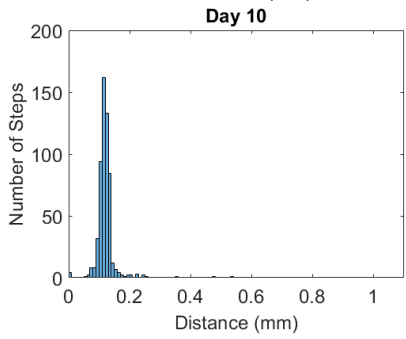

# Participant #4

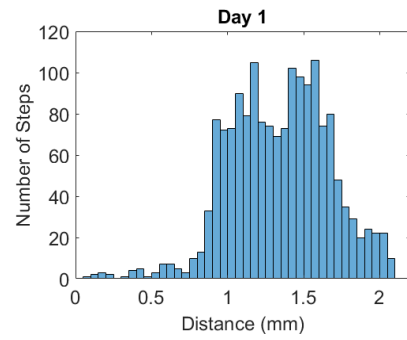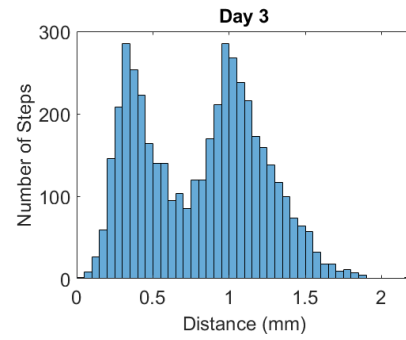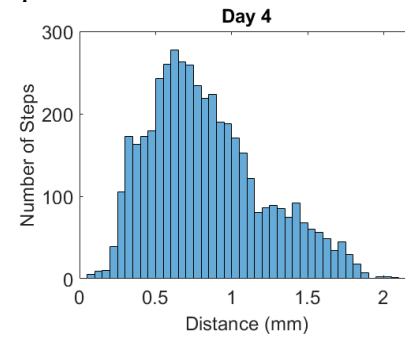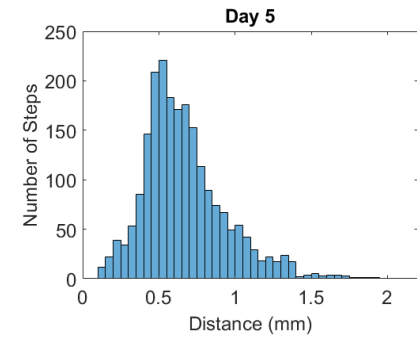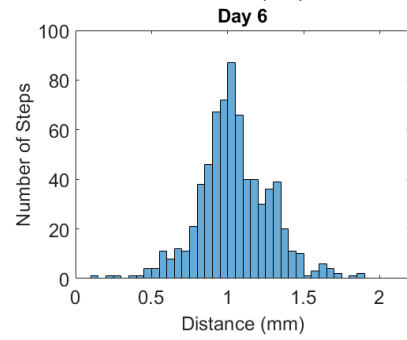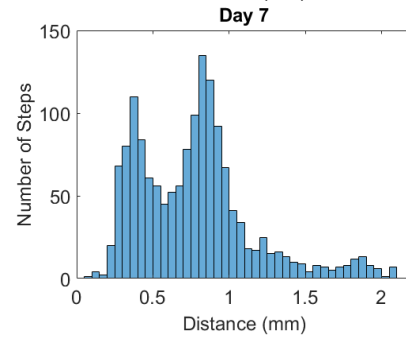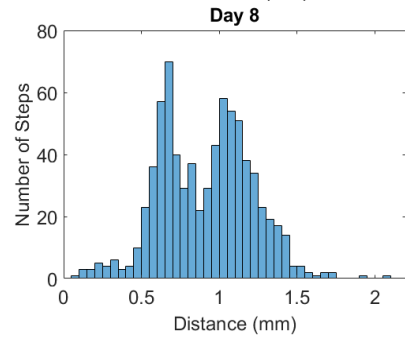

Supplement: Supplementary file 6 [file Image2.pdf]
