# Supplementary material for: Distal weight bearing in transtibial prosthesis users wearing pin suspension
Source: Front Rehabil Sci. 2023 Dec 21;4:1322202. doi: 10.3389/fresc.2023.1322202 (PMC10773776; doi:10.3389/fresc.2023.1322202)
Supplement: Supplementary file 7 [file Image3.pdf]

Supplementary Figure F3. Sensed Distance (y-axis) and Pin Height (x-axis).

Participant #1

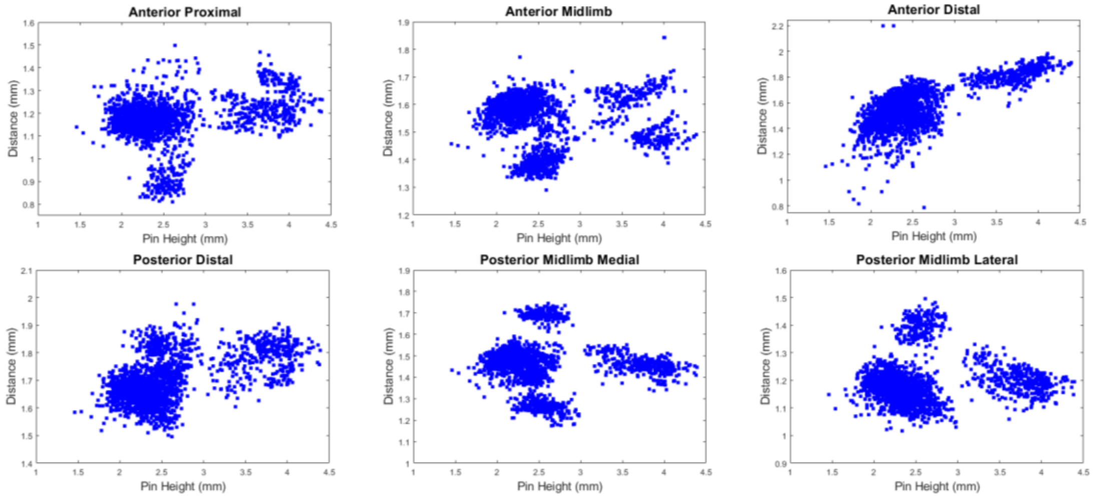

(A)

Participant #2

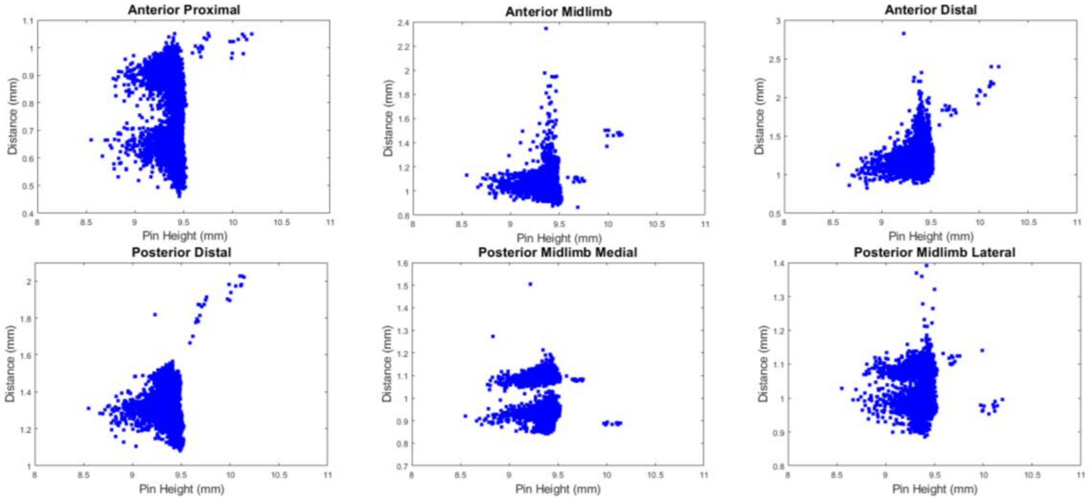

(B)

Participant #4

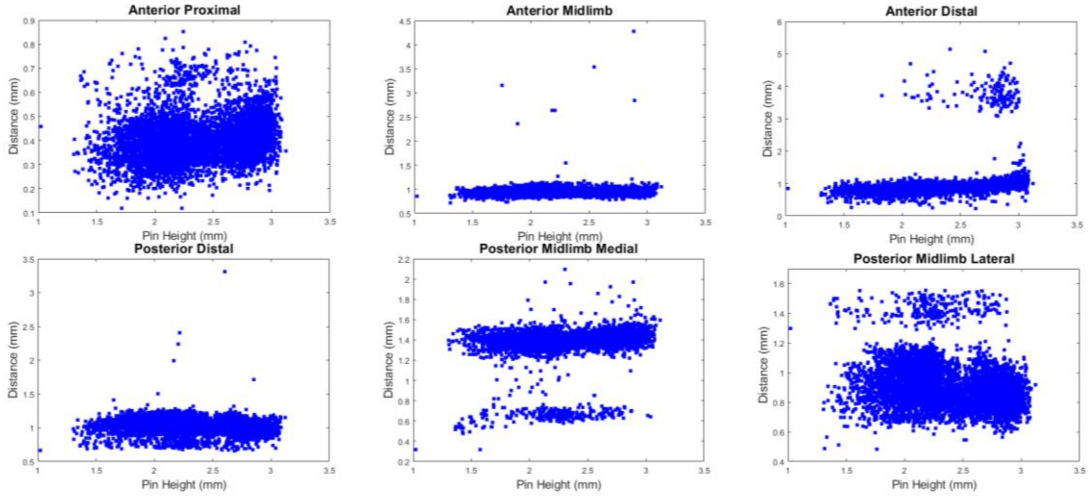

(C)
